# Supplementary material for: EnzML: multi-label prediction of enzyme classes using InterPro signatures
Source: BMC Bioinformatics. 2012 Apr 25;13:61. doi: 10.1186/1471-2105-13-61 (PMC3483700; doi:10.1186/1471-2105-13-61)
Supplement: Addtional file 5 — The Java code to format the data files, evaluate and predict. The file enzml_java_code.tar.gz contains the Java code used to format database data to ARFF and XML formats, to execute cross and train-test (jackknife) evaluations and to record evaluation results to database. More information is included in the readme.txt file and the Javadoc files. The code can be used with a MySQL database. To use a different database software, other JDBC drivers might be required. [file 1471-2105-13-61-S5.gz › java_code/enzml2011/doc/uk/ac/ed/inf/csb/taweka/mulan/learn/MulanEvaluator.html]

MulanEvaluator


---


|  |  |  |
| --- | --- | --- |
| **Overview** \| **Package** \| **Class** \| **Use** \| **Tree** \| **Deprecated** \| **Index** \| **Help** | |  |
| **PREV CLASS**   **NEXT CLASS** | **FRAMES**    **NO FRAMES**     **All Classes** |  |
| SUMMARY: NESTED \| FIELD \| CONSTR \| METHOD | DETAIL: FIELD \| CONSTR \| METHOD |  |


---


## uk.ac.ed.inf.csb.taweka.mulan.learn Class MulanEvaluator

```
java.lang.Object
  mulan.evaluation.Evaluator
      uk.ac.ed.inf.csb.taweka.mulan.learn.MulanEvaluator
```

---

``` public class MulanEvaluator extends mulan.evaluation.Evaluator ```

Class created to extract individual evaluation results (not possible from the
Mulan class Evaluator, because only averaged results are returned).

**Version:**
:   8 Jun 2010

**Author:**
:   Luna De Ferrari luna.deferrari-at-ed.ac.uk

---

**Field Summary**

**Fields inherited from class mulan.evaluation.Evaluator**

`DEFAULTFOLDS`


**Constructor Summary**

`MulanEvaluator(java.lang.String arffFilePath,
java.lang.String xmlFilePath,
mulan.classifier.MultiLabelLearner learner)`


| **Method Summary** | |
| --- | --- |
| `mulan.evaluation.Evaluation[]` | `crossValidate(mulan.classifier.MultiLabelLearner learner, mulan.data.MultiLabelInstances mlDataSet, int numFolds, int seed)`             Evaluates a `MultiLabelLearner` via cross-validation on given data set with defined number of folds. |
| `java.lang.String` | `evaluate(mulan.classifier.MultiLabelLearner learner, mulan.data.MultiLabelInstances mlDataSet, int numFolds)`             Evaluates (runs a cross validation) |
| `mulan.classifier.MultiLabelLearner` | `getAlgorithm()` |
| `java.lang.String` | `getArffFilePath()` |
| `java.lang.String` | `getEndTime()` |
| `mulan.evaluation.Evaluation[]` | `getEvaluationResults()` |
| `java.lang.String` | `getStartTime()` |
| `java.lang.String` | `getXmlFilePath()` |

**Methods inherited from class mulan.evaluation.Evaluator**

`crossValidate, crossValidate, crossValidate, crossValidate, evaluate, evaluate`

**Methods inherited from class java.lang.Object**

`equals, getClass, hashCode, notify, notifyAll, toString, wait, wait, wait`

**Constructor Detail**

### MulanEvaluator

```
public MulanEvaluator(java.lang.String arffFilePath,
                      java.lang.String xmlFilePath,
                      mulan.classifier.MultiLabelLearner learner)
```

**Parameters:**: `arffFilePath` -: `xmlFilePath` -: `learner` -


**Method Detail**

### crossValidate

```
public mulan.evaluation.Evaluation[] crossValidate(mulan.classifier.MultiLabelLearner learner,
                                                   mulan.data.MultiLabelInstances mlDataSet,
                                                   int numFolds,
                                                   int seed)
                                            throws java.lang.Exception
```

:   Evaluates a `MultiLabelLearner` via cross-validation on given data
    set with defined number of folds. The specified number of folds has to be
    at least two. If negative value is specified, the used number of folds is
    equal to number of instances in the data set.

    :   **Parameters:**: `learner` - the learner to be evaluated via cross-validation: `mlDataSet` - the multi-label data set for cross-validation: `numFolds` - the number of folds to be used **Returns:**: the evaluation results (one Evaluation per fold combination) **Throws:**: `java.lang.IllegalArgumentException` - if either of learner or data set parameters is null: `java.lang.IllegalArgumentException` - if number of folds is invalid: `java.lang.Exception`

---


### evaluate

```
public java.lang.String evaluate(mulan.classifier.MultiLabelLearner learner,
                                 mulan.data.MultiLabelInstances mlDataSet,
                                 int numFolds)
```

:   Evaluates (runs a cross validation)

    :   **Parameters:**: `learner` -: `mlDataSet` -: `numFolds` - **Returns:**: the timestamp of the end of the computation

---


### getAlgorithm

```
public mulan.classifier.MultiLabelLearner getAlgorithm()
```

---


### getArffFilePath

```
public java.lang.String getArffFilePath()
```

---


### getEndTime

```
public java.lang.String getEndTime()
```

---


### getEvaluationResults

```
public mulan.evaluation.Evaluation[] getEvaluationResults()
```

---


### getStartTime

```
public java.lang.String getStartTime()
```

---


### getXmlFilePath

```
public java.lang.String getXmlFilePath()
```


---


|  |  |  |
| --- | --- | --- |
| **Overview** \| **Package** \| **Class** \| **Use** \| **Tree** \| **Deprecated** \| **Index** \| **Help** | |  |
| **PREV CLASS**   **NEXT CLASS** | **FRAMES**    **NO FRAMES**     **All Classes** |  |
| SUMMARY: NESTED \| FIELD \| CONSTR \| METHOD | DETAIL: FIELD \| CONSTR \| METHOD |  |


---
